# Hangeshashinto for the prevention of oral mucositis in patients receiving chemotherapy: a systematic review and meta-analysis

**DOI:** 10.1007/s00520-026-10975-6

**Published:** 2026-07-09

**Authors:** Mitsuru Ishizuka, Norisuke Shibuya, Hiroyuki Hachiya, Yusuke Nishi, Takahiro Kono, Masashi Takayanagi, Tetsutaro Nemoto, Keisuke Ihara, Takatoshi Nakamura, Tsunekazu Mizushima

**Affiliations:** Department of Colorectal Surgery, Dokkyo Medical University, 880 Kitakobayashi, Mibu, Tochigi 321-0293 Japan

**Keywords:** Chemotherapy, Hangeshashinto, Herbal medicine, Meta-analysis, Oral mucositis

## Abstract

**Background:**

Among several adverse events induced by chemotherapy for patients with far advanced and unresectable cancer, oral mucositis is one that reduces patient quality of life.

**Objective:**

To evaluate the preventive effects of hangeshashinto on oral mucositis induced by chemotherapy.

**Methods:**

We performed a comprehensive electronic literature search (PubMed, the Web of Science, and CENTRAL) up to March 2024 to identify studies showing the efficacy of hangeshashinto administration for preventing oral mucositis in patients receiving chemotherapy. To integrate the individual preventive effect of hangeshashinto, a meta-analysis was performed using random-effects models to calculate the risk ratio and 95% confidence interval, and heterogeneity was analyzed using *I*^2^ statistics. In this analysis, oral mucositis was defined as more than grade 1 mucositis.

**Results:**

This meta-analysis included six studies (five randomized controlled trials and one case-matched study) involving 293 patients with cancer receiving chemotherapy. Among 141 patients who received hangeshashinto during chemotherapy, 65 (46.1%) developed oral mucositis; in contrast, among 152 patients who did not receive hangeshashinto or received a placebo, 78 (51.3%) developed oral mucositis. The meta-analysis suggested that hangeshashinto may reduce the risk of oral mucositis (risk ratio = 0.86, 95% confidence interval = 0.73–1.00, *P* = 0.05, *I*^2^ = 0%) compared with no hangeshashinto or placebo.

**Conclusions:**

The results of this meta-analysis suggest that hangeshashinto may have a preventive effect against chemotherapy-induced oral mucositis. However, further well-designed randomized controlled trials are required to confirm its preventive efficacy.

**Supplementary Information:**

The online version contains supplementary material available at 10.1007/s00520-026-10975-6.

## Introduction

Hangeshashinto (HST) is a well-known traditional Japanese herbal (*Kampo*) medicine administered to improve gastrointestinal symptoms, such as heartburn, gastritis, stomatitis, and diarrhea. It consists of seven extracted crude drugs (Coptidis Rhizome, Ginseng Radix, Glycyrrhizae Radix, Pinelliae Tuber, Scutellariae Radix, Zingiberis Rhizoma Processum, and Zizyphi Fructus) and has multiple pharmacological effects [1, 2]. Because the medicinal effects of HST have been established through the refinement of a delicate balance of these herbal ingredients over many centuries, HST has not yet been entirely accepted by all modern physicians, including those in both Eastern and Western countries.

However, accumulating evidence suggests that Japanese herbal medicines have beneficial effects on cancer chemotherapy–induced adverse events [3–5]. For patients with far advanced and unresectable cancer, oral mucositis (OM) [6–8] is a treatment-related adverse event that reduces quality of life [9, 10]. In preclinical studies carried out over the last decade, HST directly inhibited prostaglandin E2 production in human gingival fibroblasts and reduced the prostaglandin E2 content in the colon, resulting in the improvement of inflammatory damage in several animal models of diarrhea induced by anticancer drugs, cholera toxin, or castor oil [11–13]. In addition, in several studies, HST exerted strong antibacterial effects [14] and attenuated oral chemotherapy–induced stomatitis in rats [15]. HST also increased the viability and invasion of epidermal keratinocytes and fibroblasts [16, 17]. Similarly, HST reduced the severity of radiation-induced OM in an animal model by suppressing the inflammatory response [18, 19]. Further, in a clinical study, topical administration of HST significantly reduced grade 3/4 mucositis in 13 of 14 patients with chemotherapy-induced OM, and this therapeutic effect was considered to occur via down-regulation of pro-inflammatory prostaglandins in the cyclooxygenase pathway [20].

Recently, many randomized controlled trials (RCTs) [21–25], including a case-matched study [26], have been performed to investigate the preventive effects of HST on OM induced by chemotherapy. Although two meta-analyses investigated the preventive effect of HST on chemotherapy-induced diarrhea [27, 28], only one has explored its potential preventive effect on OM for patients receiving chemotherapy [29].

Therefore, we performed a meta-analysis of RCTs and a case-matched study to investigate whether the administration of HST prevents the development of OM in patients with cancer receiving chemotherapy.

## Methods

### Search strategy

A systematic literature search of CENTRAL, PubMed, and the Web of Science was conducted on April 24, 2024, and included publications indexed by March 31, 2024. The search was focused on articles reporting results of RCTs, case-control studies, matched case-control studies, and propensity score–matched cohort studies. The search terms included “hangeshashinto,” “kampo,” “anticancer,” “stomatitis,” and related Medical Subject Heading (MeSH) terms. For those publications identified as potentially relevant, complete articles were retrieved and evaluated for inclusion. References from the relevant articles were searched manually for additional studies. The entire search strategy is available in the supplemental files.

The meta-analysis and search strategy followed the Preferred Reporting Items for Systematic Reviews and Meta-Analyses (PRISMA) 2020 guidelines [30] and the PRISMA 2020 flow diagram for new systematic reviews, which included searches of databases and registers only. The Patients, Intervention, Comparison, Outcome (PICO) criteria for this study were the following: patients receiving chemotherapy, HST, without HST or placebo, and incidence of OM.

### Inclusion and exclusion criteria

The inclusion criteria were as follows: (1) studies that provided data suitable for the evaluation of the risk of OM; (2) studies that provided the sample size and other appropriate data; (3) studies that provided data allowing calculation of the risk ratio (RR) or standardized incidence ratio with 95% confidence intervals (CIs); (4) articles written in English; and (5) randomized or case-matched studies.

The exclusion criteria were as follows: (1) non-reporting of predefined outcomes for the two groups (patients with chemotherapy who received and did not receive HST or a placebo); and (2) letters, commentaries, correspondences, editorials, and reviews. Finally, we excluded articles that overlapped considerably with other articles regarding authors, centers, and participants.

### Study selection and data extraction

Two authors (M.I. and N.S.) independently performed full-text reviews based on the inclusion and exclusion criteria and the PICO criteria. Any disagreements were resolved by discussion and consensus. The same two authors also independently extracted the following information from each eligible article: the first author’s name, the year of publication, the country in which the study was conducted, the study design, the number of patients who received chemotherapy, the sample size, and the incidence of OM. If the necessary data could not be extracted from the publication, we contacted the original authors directly whenever possible.

### Definition of oral mucositis

The severity of OM was determined using the National Cancer Institute Common Terminology Criteria for Adverse Events version 5.0: Grade (G)1, asymptomatic or mild symptoms, intervention not indicated; G2, moderate pain or ulcer that does not interfere with oral intake, modified diet indicated; G3, severe pain interfering with oral intake; G4, life-threatening consequences, urgent intervention indicated; and G5, death.

### Risk of bias assessment

Two authors (M.I. and N.S.) independently assessed the risk of bias for each study. Bias was assessed using the ROBINS-1 tool for non-randomized studies [31] and the Cochrane Risk of Bias tool for randomized controlled studies [32]. Any discrepancy between the two authors was resolved by discussion and mutual agreement. If necessary, a third author (H.H.) was involved.

### GRADE assessment

We evaluated the level of evidence using the Grades of Recommendation, Assessment, Development, and Evaluation (GRADE) approach [33]. In addition, the GRADE profiler, version 3.6, was used to create the evidence profile. The evaluation standards included study limitations, inconsistencies of the results, indirectness, imprecision, and publishing biases.

The GRADE system classifies the quality of evidence into four levels: “high,” “moderate,” “low,” and “very low.” Two authors (M.I. and N.S.) completed data extraction and quality assessment; any contradictions were resolved with the input of the third author (H.H.).

### Data synthesis and statistical analysis

This meta-analysis used Review Manager (ver. 5.3) for Windows (downloaded from http://ims.cochrane.org/revman/download).

Dichotomous variables were analyzed by assessing the RR of OM in patients treated with HST compared with those not treated with HST or treated with a placebo as a control group, along with the 95% CI. A total RR of less than 1 was defined as favoring patients with chemotherapy who received HST in reducing the risk of OM.

Statistical heterogeneity was calculated using the *I*^2^ statistics, which determined the proportion of total variation across studies due to heterogeneity rather than chance. We interpreted *I*^2^ values of 0% to 50% to indicate low heterogeneity, > 50% to 75% to indicate moderate heterogeneity, and > 75% to 100% to indicate high heterogeneity.

The weight of each study was shown in ordered forest plots. A *P* value < 0.05 was considered to indicate statistical significance.

Funnel plots were created to illustrate the weight of each publication bias.

Ethical approval was not required because this was a meta-analysis of previously published literature.

## Results

### Study identification and eligibility

**Fig. 1:**
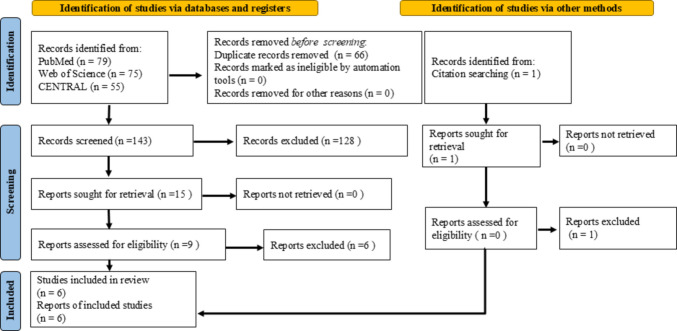
Search strategy and identification of the studies included in the analysis

An electronic search yielded 209 articles, of which 66 were regarded as duplicates based on a title search. Among the remaining articles, 128 were excluded by title/abstract review based on the selection criteria and the PICO criteria. The remaining 15 articles were screened by full-text review, after which 6 studies, including 293 patients, were regarded as suitable for inclusion in the data synthesis. The selection process for exclusion is demonstrated in Fig. 1.

### Characteristics of included studies

All studies were published from 2014 to 2023 and originated in Japan. Among the 6 studies, 2 were multi-center studies [21, 24], and 4 were single-center studies [22, 23, 25, 26]. These 6 studies comprised 5 RCTs [21–25] and one case-matched study [26]. Two of the 6 studies involved patients undergoing surgery for gastric [24] or colon [21] cancer. Among the remaining 4 studies, 2 involved patients undergoing surgery for esophageal cancer [22, 25], and 2 involved patients undergoing surgery for head and neck cancer [23, 26]. Among the 2 studies in head and neck cancer, one targeted patients subjected to radiation therapy with concomitant administration of anticancer drugs [26]. Among all 6 studies, 3 used a placebo [21, 23, 24], and 3 did not [22, 25, 26]; 2 included radiation therapy [25, 26], and 4 did not [21–24]. All studies defined chemotherapy-induced OM as OM more than G1. Five studies administered HST 2.5 g × 3/day [21–25], and the remaining one administered HST 0.625 g × 3/day [26]. Four of the 6 included studies administered HST orally with a rinse for the patient’s oral cavity [21, 23–25]. Among the remaining 2 studies, one administered HST as sorbet [22], and one administered HST as a frozen preparation for cryotherapy [26]. Two of the 6 studies administered HST for 2 weeks [21, 23], and one administered HST for 8 weeks [25]. In the remaining studies, HST was administered from 2 to 6 weeks [24], and 2 had no data on the duration of administration [22, 26]. The essential characteristics of the 6 included studies are shown in Table 1.

**Table 1:** Summary of included studies

Baseline characteristics											
Study	Year	Country	Number of centers	Design	Type of cancer	Placebo	RT	Grade of OM	Dose	Method	Term
Aoyama et al. [24]	2014	Japan	Multi	RCT	Gastric	+	−	≥ 2	2.5 g × 3/day	OI	2–6 weeks
Matsuda et al. [21]	2015	Japan	Multi	RCT	Colorectal	+	−	≥ 2	2.5 g × 3/day	OI	2 weeks
Moriyama et al. [22]	2018	Japan	Single	RCT	Esophageal	−	−	≥ 2	2.5 g × 3/day	Sorbet	Undetermined
Takahashi et al. [25]	2018	Japan	Single	RCT	Esophageal	−	+	≥ 2	2.5 g × 3/day	OI	8 weeks
Taira et al. [23]	2020	Japan	Single	RCT	Head and neck	+	−	≥ 2	2.5 g × 3/day	OI	2 weeks
Kato et al. [26]	2023	Japan	Single	Matched study	Head and neck	−	+	≥ 2	0.625 g/day	Frozen	Undetermined

*RCT* randomized controlled trial, *OI* oral intake, *OM* oral mucositis, *RT* radiation therapy

### Risk of bias assessment of included studies

Tables 2 and 3 summarize the quality assessment of the one non-randomized study [26] and the 5 RCTs [21–25] included in the analysis.

**Table 2:** Risk of bias assessment in one non-randomized controlled study

Author	D1	D2	D3	D4	D5	D6	D7	Overall
Kato et al. [26]	−	X	−	X	+	+	+	−

*N/A*, not available

Domains: D1: bias due to confounding, D2: bias due to the selection of participants, D3: bias in the classification of interventions, D4: bias due to deviations from intended interventions, D5: bias due to missing data, D6: bias in measurement of outcome, D7: bias in the selection of the reported result

Judgment: X: serious, −: moderate, +: low

**Table 3:** Risk of bias assessment in 5 randomized controlled studies

Author	D1	D2	D3	D4	D5	Overall
Aoyama et al. [24]	+	+	+	+	+	+
Matsuda et al. [21]	+	+	+	+	+	+
Moriyama et al. [22]	−	+	+	−	−	−
Takahashi et al. [25]	−	+	+	X	X	X
Taira et al. [23]	−	+	−	−	−	−

Domains: D1: bias arising from the randomization process, D2: bias due to deviations from the intended intervention, D3: bias due to missing outcome data, D4: bias in measurement of the outcome, D5: bias in the selection of the reported result

Judgment: X: serious, −: moderate, +: low

The results of the risk of bias assessment indicated that the case-matched study had a moderate risk of bias [26]. Among the 5 RCTs, two had a low risk of bias [21, 24], two had a moderate risk of bias [22, 23], and one had a severe risk of bias [25].

### GRADE assessment of the risk of OM

A summary of the GRADE assessment of the risk of OM is provided below.

The overall risk analysis of OM was regarded as moderate quality. The importance level was regarded as “important” because even though the case-matched study might have included hidden biases compared with the 5 RCTs, the results revealed that the RR was 0.86 and the 95% CI was 0.73–1.00.

### Association between the administration of HST and the incidence of OM

**Fig. 2:**
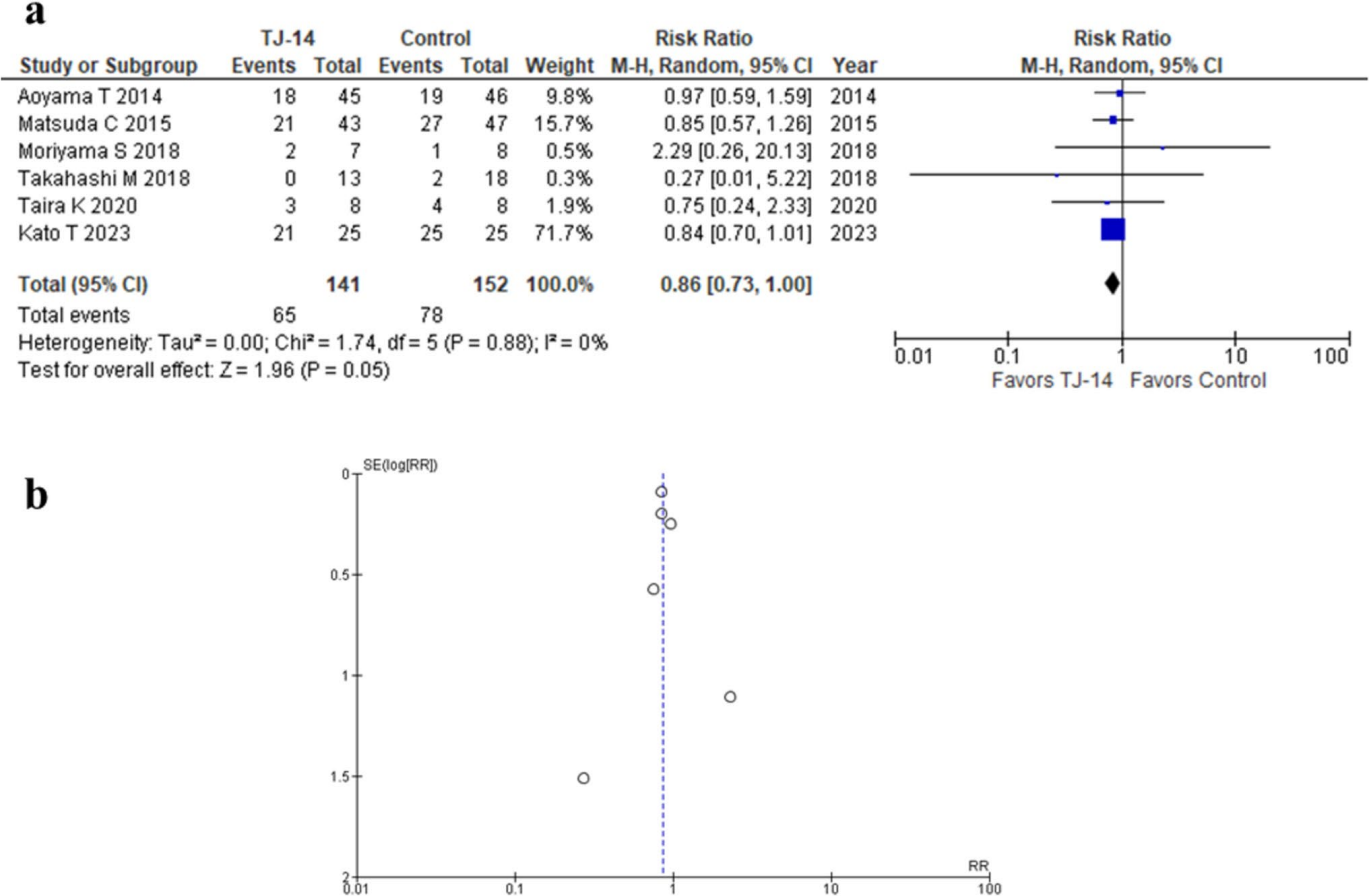
All studies included a forest plot analysis of the overall risk of chemotherapy-induced OM in patients who did or did not receive HST. **a** Forest plot analysis of the overall risk of chemotherapy-induced OM from all included studies. **b** Funnel plot analysis using integrated data. CI, confidence interval; df, degrees of freedom; M-H, Mantel-Haenszel

Table 4 summarizes the incidence of chemotherapy-induced OM in the included studies. Data on the overall incidence of OM were available for all six studies. A total of 293 patients were included in the analysis. Among 141 patients who received HST during chemotherapy, 65 (46.1%) developed OM, whereas among 152 patients who did not receive HST or received a placebo, 78 (51.3%) developed OM. The overall incidence of chemotherapy-induced OM tended to be lower in patients who received HST than in those who did not receive HST or received a placebo (RR, 0.86; 95% CI, 0.73–1.00; *P* = 0.05; *I*^2^ = 0%) (Fig. 2a). These findings suggest that HST may reduce the risk of chemotherapy-induced OM; however, the confidence interval reached the null value and therefore the findings should be interpreted with caution. Funnel plot assessment did not reveal obvious asymmetry; however, publication bias cannot be excluded because only six studies were included (Fig. 2b).

**Table 4:** Relationship between administration of Hangeshasinto and oral mucositis

Study	Hangeshashinto +			Hangeshashinto −		
	Total	OM+	OM-	Total	OM+	OM-
Aoyama et al. [24]	45	18	27	46	19	27
Matsuda et al. [21]	43	21	22	47	27	20
Moriyama et al. [22]	7	2	5	8	1	7
Takahashi et al. [25]	13	0	13	18	2	16
Taira et al. [23]	8	3	5	8	4	4
Kato et al. [26]	25	21	4	25	25	0
Total	141 (100%)	65 (46.1%)	76 (53.9%)	152 (100%)	78 (51.3%)	74 (48.7%)

*OM*, oral mucositis

## Discussion

Although our previous meta-analysis first demonstrated the efficacy of herbal medicine (Japanese Kampo medicine) for reducing the risk of postoperative ileus [34], some criticism arose regarding the evidence level because the study did not formally assess the risk of bias [35, 36]. To decrease biases, including publication bias, only RCTs and a case-matched study were chosen in this analysis. Because this meta-analysis revealed an *I*^2^ value of 0%, indicating low statistical heterogeneity among the included studies, the pooled results suggested a favorable trend toward a reduced risk of OM in patients receiving chemotherapy.

Moreover, to evaluate the risk of bias of the studies, first, we performed a quality assessment of the one non-randomized study [26] and the 5 RCTs [21–25] included in the analysis. The quality assessment indicated that these studies were suitable for inclusion in the meta-analysis. Second, we performed a GRADE assessment of the risk of chemotherapy-induced OM. The results demonstrated that the overall risk analysis of chemotherapy-induced OM was considered moderate quality, and the importance level was regarded as “important.”

In addition, our other meta-analysis integrating two of the studies included in the current meta-analysis [21, 24] showed no significant difference in the treatment effect of HST on chemotherapy-induced OM between patients with and without HST treatment (RR, 0.81; 95% CI, 0.56–1.17; *P* = 0.26; *I*^2^ = 0%). Therefore, although HST may have a preventive effect on chemotherapy-induced OM, evidence supporting a treatment effect remains insufficient. However, because this result was based on only two RCTs, further studies are required before any conclusion regarding a therapeutic effect can be drawn. Both studies [21, 24] showed a trend toward improved treatment outcomes in patients who received HST compared with those who did not.

On the other hand, one study that investigated the efficacy of frozen HST for radiation-induced OM in patients receiving concomitant administration of anticancer drugs revealed that frozen HST significantly delayed the onset of OM (16.1 ± 10.1 days vs 9.5 ± 4.9 days, *P* < 0.01) and diminished the duration of radiation-induced OM (33.3 ± 14.6 days vs 49.2 ± 11.1 days, *P* < 0.01), respectively [26]. Therefore, additional RCTs are required to clarify the effects of this treatment on chemotherapy-induced OM.

Because HST consists of several ingredients, it has several treatment effects for patients with gastrointestinal symptoms. Although a recent meta-analysis disclosed that HST did not reduce the incidence of irinotecan-induced mild diarrhea (RR 1.35, 95% CI 0.87–2.09, *P* = 0.18; low-quality evidence), in a subgroup analysis compared with no treatment, the HST group had a significantly lower incidence of irinotecan-induced severe diarrhea (RR 0.17, 95% CI 0.03–0.88, *P* = 0.03; low-quality evidence) [28].

Because one RCT among the included studies investigated the efficacy of HST for the prevention of OM in patients with colorectal cancer who received chemotherapy, including an irinotecan regimen [21], HST may be a promising supportive-care option for patients receiving irinotecan-based chemotherapy.

This study has some limitations. First, a limited number of patients were included based on race and location; all six studies were from Japan. Second, one of the studies was a case-matched study [26]. Although the case-matched study had well-balanced background factors, it was based on a retrospective analysis. Thus, the possibility of hidden biases cannot be entirely ruled out. Third, in two of the six studies, HST was administered in a frozen form, such as sorbet [22] or frozen [26] preparations. This method of administration raises the possibility that, beyond the pharmacological effects of HST itself, the formulation may have had an additional preventive effect against chemotherapy-induced OM. Fourth, an additional limitation of this study is that two [25, 26] of the six included studies involved patients receiving radiotherapy. Because radiotherapy is a well-established risk factor for oral mucositis, its inclusion may have influenced the pooled preventive effect. However, in a sensitivity analysis excluding the two radiotherapy-related studies [25, 26], the pooled risk ratio changed only modestly from 0.86 (95% CI, 0.73–1.00) to 0.90 (95% CI, 0.67–1.21), and the direction of the preventive effect remained unchanged. These findings suggest that the overall results were relatively robust to the inclusion of radiotherapy-related studies, although the specific contribution of radiotherapy to the preventive effect of HST could not be determined. Finally, several types of doses and treatment durations were used in these studies. Although, among the six studies, only one administered 0.625 g/day of HST [26], there was a broad range of treatment durations, from 2 to 8 weeks, in patients undergoing chemotherapy. Although 2 weeks of administration of HST [21, 23] could be sufficient to prevent chemotherapy-induced OM, it might be insufficient for the treatment of chemotherapy-induced OM. In the future, it will be necessary to examine not only the prevention of chemotherapy-induced OM but also the optimal duration of HST administration required to treat chemotherapy-induced OM effectively.

Furthermore, clinical heterogeneity should be considered when interpreting the present findings. The included studies involved different cancer types, including gastric [24], colorectal [21], esophageal [22, 25], and head and neck cancers [23, 26], and various chemotherapy regimens were used across the studies. Differences in patient populations, treatment protocols, and supportive-care practices may have influenced the observed treatment effects. Therefore, although the pooled analysis suggested a potential preventive effect of HST against chemotherapy-induced OM, the results should be interpreted with caution. Future large-scale RCTs using more homogeneous patient populations and treatment regimens are warranted to confirm these findings.

Despite these limitations, this is the first meta-analysis to evaluate the preventive effect of HST on chemotherapy-induced OM using predominantly randomized evidence and formal risk-of-bias assessment.

In conclusion, this meta-analysis suggests that HST may have a preventive effect against chemotherapy-induced OM in patients receiving chemotherapy. However, further well-designed RCTs are required to confirm its efficacy.

## Supplementary Information

Below is the link to the electronic supplementary material.

ESM 1 (DOCX 18.9 KB)

ESM 2 (DOCX 28.5 KB)

## Data Availability

No datasets were generated or analysed during the current study.

## Abbreviations

CI: Confidence interval
GRADE: Grading of Recommendations Assessment, Development, and Evaluation
HST: Hangeshashinto
OI: Oral intake
OM: Oral mucositis
PICO: Patients, intervention, comparison, outcome
PRISMA: Preferred Reporting Items for Systematic Reviews and Meta-Analyses
RCT: Randomized controlled trial
RR: Risk ratio
RT: Radiation therapy
