# Supplementary material for: Hangeshashinto for the prevention of oral mucositis in patients receiving chemotherapy: a systematic review and meta-analysis
Source: Support Care Cancer. 2026 Jul 9;34(8):749. doi: 10.1007/s00520-026-10975-6 (PMC13350161; doi:10.1007/s00520-026-10975-6)
Supplement: Supplementary file 1 — (DOCX 18.9 KB) [file 520_2026_10975_MOESM1_ESM.docx]

Search results of literature databases

The last running date of the literature search is March 31, 2024.

Search strings & results

1. PubMed (MEDLINE & PMC)

#1: "antineoplastic agents"[MeSH Terms] OR "neoplasms"[MeSH Terms] OR "neoplasm*"[Title/Abstract] OR "tumo*"[Title/Abstract] OR "cancer*"[Title/Abstract] OR "malignan*"[Title/Abstract] OR "chemotherap*"[Title/Abstract] OR "anti tumo*"[Title/Abstract] OR "antineoplastic*"[Title/Abstract] OR "anti neoplastic*"[Title/Abstract] OR "anti cancer*"[Title/Abstract]

#2: "hange-shashinto"[Supplementary Concept] OR "banxiaxiexintang decoction"[Supplementary Concept] OR "hangeshashinto"[Title/Abstract] OR "hange-shashin"[Title/Abstract] OR "banxiaxiexin"[Title/Abstract] OR "banxia-xiexin"[Title/Abstract] OR "ban-xia-xie-xin"[Title/Abstract] OR "banha-sasim-tang"[Title/Abstract] OR "banhasasim-tang"[Title/Abstract] OR "TJ-14"[Title/Abstract] OR (("Pinellia"[MeSH Terms] OR "Pinellia"[Title/Abstract]) AND ("heart"[Title/Abstract] OR "decoction"[Title/Abstract] OR "drain"[Title/Abstract])) OR "medicine, chinese traditional"[MeSH Terms:noexp] OR "medicine, kampo"[MeSH Terms] OR "medicine, korean traditional"[MeSH Terms] OR "kampo"[Title/Abstract] OR "traditional chinese medicine"[Title/Abstract] OR "chinese traditional medicine"[Title/Abstract] OR "chinese herbal medicine"[Title/Abstract] OR "traditional korean medicine"[Title/Abstract] OR "korean traditional medicine"[Title/Abstract] OR "korean herbal medicine"[Title/Abstract] OR "drugs, chinese herbal"[MeSH Terms]

#3: "stomatitis"[MeSH Terms] OR "stomatiti*"[Title/Abstract] OR "oromucositi*"[Title/Abstract] OR "oral inflammation"[Title/Abstract] OR "oral mucositi*"[Title/Abstract] OR "Pyostomatitis"[Title/Abstract] OR "plasma cell orificial mucositis"[Title/Abstract] OR "plasma cell mucositis"[Title/Abstract]

#4: #1 and #2 and #3

#5: "0000/01/01"[Date - Create]: "2024/03/31"[Date - Create]

#6: #4 AND #5

1. Web of Science (Science Citation Index Expanded(SCIE))

#1: TS=neoplasm* OR TS=tumo* OR TS=cancer* OR TS=malignan* OR TS=chemotherap* OR TS="anti tumo*" OR TS=antineoplastic* OR TS="anti neoplastic*" OR TS="anti cancer*"

#2: (((((TS=chinese OR TS=Korean) AND (TS=Herbal OR TS=traditional)) OR

(TS=Pinellia AND (TS=heart OR TS=decoction OR TS=drain)) OR TS=hangeshashinto OR TS=hange-shashin OR TS=banxiaxiexin OR TS=banxia-xiexin OR TS=ban-xia-xie-xin OR TS=banha-sasim-tang OR TS=banhasasim-tang OR TS=TJ-14 OR TS=kampo)))

#3: TS=stomatiti* OR TS=oromucositi* OR TS="oral inflammation" OR TS="oral mucositi*" OR TS=Pyostomatitis OR TS="plasma cell orificial mucositis" OR TS="plasma cell mucositis"

#4 #1 and #2 and #3

#5 #4 with custom index date from January 1, 1973, to March 31, 2024

1. Cochrane Library (Cochrane Central Register of Controlled Trials(CENTRAL))

#1: [mh "antineoplastic agents"] OR [mh neoplasms] OR neoplasm*:ti,ab,kw OR tumo*:ti,ab,kw OR cancer*:ti,ab,kw OR malignan*:ti,ab,kw OR chemotherap*:ti,ab,kw OR ("anti" NEXT tumo*):ti,ab,kw OR antineoplastic*:ti,ab,kw OR ("anti" NEXT neoplastic*):ti,ab,kw OR ("anti" NEXT cancer*):ti,ab,kw

#2: hangeshashinto:ti,ab,kw OR hange-shashin:ti,ab,kw OR banxiaxiexin:ti,ab,kw OR banxia-xiexin:ti,ab,kw OR ban-xia-xie-xin:ti,ab,kw OR banha-sasim-tang:ti,ab,kw OR banhasasim-tang:ti,ab,kw OR TJ-14:ti,ab,kw OR (([mh Pinellia] OR Pinellia:ti,ab,kw) AND (heart:ti,ab,kw OR decoction:ti,ab,kw OR drain:ti,ab,kw)) OR [mh ^"medicine, chinese traditional"] OR [mh "medicine, kampo"] OR [mh "medicine, korean traditional"] OR kampo:ti,ab,kw OR "traditional chinese medicine":ti,ab,kw OR "chinese traditional medicine":ti,ab,kw OR "chinese herbal medicine":ti,ab,kw OR "traditional korean medicine":ti,ab,kw OR "korean traditional medicine":ti,ab,kw OR "korean herbal medicine":ti,ab,kw OR [mh "drugs, chinese herbal"]

#3: [mh stomatitis] OR stomatiti*:ti,ab,kw OR oromucositi*:ti,ab,kw OR "oral inflammation":ti,ab,kw OR ("oral" NEXT mucositi*):ti,ab,kw OR Pyostomatitis:ti,ab,kw OR "plasma cell orificial mucositis":ti,ab,kw OR "plasma cell mucositis":ti,ab,kw

#4: #3 with Date added to CENTRAL trials database January 1 0000 to March 31, 2024
