# Supplementary material for: Hangeshashinto for the prevention of oral mucositis in patients receiving chemotherapy: a systematic review and meta-analysis
Source: Support Care Cancer. 2026 Jul 9;34(8):749. doi: 10.1007/s00520-026-10975-6 (PMC13350161; doi:10.1007/s00520-026-10975-6)
Supplement: Supplementary file 2 — (DOCX 28.5 KB) [file 520_2026_10975_MOESM2_ESM.docx]

PRISMA 2020 Checklist

(Systematic Review / Meta-analysis)

Section / Topic Item # Checklist Item Location in Manuscript (Page)

TITLE

Title 1 Identify the report as a systematic review. ___1___

ABSTRACT

Abstract 2 See PRISMA 2020 for abstracts checklist. ___3___

INTRODUCTION

Rationale 3 Describe the rationale for the review. ___6___

Objectives 4 Provide an explicit statement of objectives. ___7___

METHODS

Eligibility criteria 5 Specify inclusion and exclusion criteria. ____8__

Information sources 6 Specify all sources (databases, registers, etc.). ___8___

Search strategy 7 Present full search strategies for all databases. ____8__

Selection process 8 Specify methods used to select studies. ____8__

Data collection 9 Specify methods used to collect data. ___8___

Data items 10 List and define all variables for which data were sought. ____8__

Study risk of bias 11 Specify methods used to assess risk of bias. ___9___

Effect measures 12 Specify effect measures used. ____9__

METHODS – SYNTHESIS

Synthesis methods 13a Describe processes used to decide study eligibility for synthesis. ___8___

13b Describe methods required to prepare data for synthesis. __8____

13c Describe methods used to tabulate or visually display results. ____8__

13d Describe methods used for synthesis (e.g., meta-analysis model). ___8___

13e Describe methods used to explore heterogeneity. ___10___

13f Describe methods used to assess robustness (sensitivity analyses). ___10___

Reporting bias 14 Describe methods to assess reporting bias. __9____

Certainty assessment 15 Describe methods to assess certainty of evidence (e.g., GRADE). ____10__

RESULTS

Study selection 16a Report number of studies screened, assessed, included. ____12__

16b Cite excluded studies with reasons. ___9___

Study characteristics 17 Cite and describe characteristics of included studies. __8____

Risk of bias 18 Present risk of bias for each study. ___13___

Results of studies 19 Present summary statistics and effect estimates. ____13__

Synthesis results 20a Present results of all analyses. __13____

20b Present results of heterogeneity analysis. ___13___

20c Present sensitivity analysis results. ___13___

Reporting bias 21 Present results of reporting bias assessment. ____13__

Certainty of evidence 22 Present certainty of evidence (e.g., GRADE). ___13___

DISCUSSION

Discussion 23a Provide a general interpretation of results. __15____

23b Discuss limitations of included evidence. ___15___

23c Discuss limitations of review processes. ___17___

23d Discuss implications for practice and research. _16_____

OTHER

Registration 24 Provide registration information (e.g., PROSPERO). ______

Protocol 25 Indicate where the protocol can be accessed. ______

Support 26 Describe sources of financial or non-financial support. __1____

Competing interests 27 Declare competing interests. ___1___

Availability of data 28 Provide information on data, code, and materials. ______
